# Early Identification and Linkage to Care of Persons with Chronic Hepatitis B Virus Infection — Three U.S. Sites, 2012–2014

**Published:** 2014-05-09

**Authors:** Geoff A. Beckett, Gilberto Ramirez, Aaron Vanderhoff, Kim Nichols, Sara M. Chute, David L. Wyles, Ben T. Schoenbachler, Deborah T. Bedell, Rebecca Cabral, John W. Ward

**Affiliations:** 1 Division of Viral Hepatitis, National Center for HIV/AIDS, Viral Hepatitis, STD, and TB Prevention, CDC; 2 African Services Committee; 3 Minnesota Department of Health; 4 University of California at San Diego

In the United States, an estimated 0.8–1.4 million persons are living with chronic hepatitis B virus (HBV) infection. Among these persons, as many as 70% were born in countries of Asia, Africa, or other regions where HBV is moderately or highly endemic (hepatitis B surface antigen [HBsAg] prevalence ≥2%) (1). HBV-associated cirrhosis and liver cancer are major health problems for these populations (2,3). Most persons with HBV were infected at birth or during early childhood and are asymptomatic until advanced liver disease develops. To address these concerns, CDC recommends HBsAg testing for all persons born in these areas and linkage to medical care and preventive services for those who are infected (1). In 2012, CDC awarded funds to nine sites to implement this recommendation. This report describes programs at three sites (New York, New York; Minneapolis-St. Paul, Minnesota; and San Diego, California) that conducted HBV testing, in clinical or community settings, and referred for medical evaluation and care those persons whose HBsAg test results were positive. During October 2012–March 2014, the three sites tested 4,727 persons for HBV infection; 310 (6.6%) were HBsAg-positive. Among the HBsAg-positive persons, 94% were informed of their results, 90% were counseled, 86% were referred for care, and 66% attended their scheduled first medical visit. These projects demonstrate that community-based programs can identify infected persons among populations with a high prevalence of HBV infection and refer HBsAg-positive persons for care. Individualized efforts to assist patients with accessing and receiving health-care services (“patient navigation services”) can increase the number of persons who follow up on referrals and receive recommended care.

## New York City: African Services Committee

The African Services Committee (ASC) is a community-based organization located in Harlem, serving a primarily uninsured West African immigrant population. Much of the work of the ASC is carried out by staff members speaking French and Wolof, a language widely used in Senegal and The Gambia. Community education efforts included outreach through mosques serving immigrants from Africa, visits to taxi garages to speak with drivers from West Africa, participating in French language “conference-call radio” shows, and issuing radio public service announcements. Most testing was conducted in community outreach settings. To improve testing rates, ASC established relationships with Asian-American community-based organizations to conduct testing events in more distant areas of the city. Persons found to be HBsAg-positive received patient navigation services for initial evaluation (and treatment, if required) to liver clinics and patient assistance programs in academic and public medical centers with which ASC has strong relationships. During the reporting period, ASC tested 1,732 persons (54% born in Africa, 23% in Asia, and 23% in Latin America or the Caribbean); 145 (8.4%) were HBsAg-positive, with the highest prevalence seen among 880 persons born in West Africa (11.4%). For all HBsAg-positive persons, 131 (90%) received test results, 120 (83%) were counseled, 123 (85%) were referred for medical evaluation, and 81 (56%) attended the first medical visit (Table).

## Minneapolis-St. Paul: Minnesota Department of Public Health Refugee Services

The Minnesota Refugee Health Program is responsible for assuring the initial health screening for communicable diseases among newly arrived refugees. Testing for HBsAg has been a routine screening activity for many years, but no resources were available to support counseling and referral for persons who were HBsAg-positive. In Minneapolis-St. Paul, the program partners with health departments serving Hennepin County and Ramsey County to provide refugees with HBsAg screening at their first medical visit, at a large health department clinic (Hennepin County), and at the offices of designated private providers (St. Paul-Ramsey County). With support through this CDC initiative, HBsAg-positive persons were informed of their test results at the second visit, counseled, and referred for further evaluation and medical care. Part-time workers who are fluent in Somali and in Karen, the language spoken by most of the refugees from Burma (Myanmar), played a critical role in the program. They provided education and counseling at the health clinic and patient navigation services for all HBsAg-positive refugees to ensure that those refugees were able to attend appointments made at a liver specialty clinic or with other primary care providers in the community. Navigation activities included scheduling cab rides to appointments, making follow-up calls after referral, and locating patients lost to follow-up. Among 1,800 refugees tested, of whom 84% were from Burma and Somalia, 117 (6.5%) tested HBsAg-positive. Of these, 111 (95%) received test results and were counseled, and 106 (91%) were referred for a medical evaluation, of whom all were documented to have attended a medical visit.

## San Diego: University of California at San Diego

At the University of California at San Diego site, medical specialists partner with the Asian Pacific Health Foundation and several other community-based organizations and clinics to provide HBsAg testing and referral services to a primarily Southeast Asian population. Education and testing were conducted at outreach events held in churches, temples, and festival venues, and at primary care centers serving foreign-born populations. Health profession students fluent in Tagalog played an important role in testing events in the Filipino-American community. Testing targeted specific neighborhoods in which large numbers of foreign-born persons reside. During the reporting period, 1,195 persons were tested, and 48 (4.0%) were HBsAg-positive. Testing was provided to persons born in 31 different counties; however, 67% of persons tested and 88% of persons who tested HBsAg-positive originated in either Viet Nam or the Philippines. Patient navigation services were provided by the community-based organization partner. Many of those persons who tested HBsAg-positive were referred to a gastroenterology practice with a large Asian-American patient population. To date, all 48 HBsAg-positive persons have been informed of their results and counseled, 39 (81%) were referred for medical evaluation, and 16 (33%) were documented to have attended a first medical visit.

### Discussion

A total of 4,727 persons were tested at these three sites. Most persons (91%) who were screened were from countries of intermediate or higher HBV infection prevalence, consistent with the purpose of the initiative. Two of the sites used community-based outreach to educate and test foreign-born persons, and newly arrived refugees in Minnesota were already being routinely screened for HBV at their first clinical visit. All three sites provided culturally and linguistically appropriate counseling and patient navigation services for persons who tested HBsAg-positive. A substantial proportion of persons found to be HBV-infected were informed of their test results (94%), counseled (90%), and referred for care (86%). Collaborations among community-based organizations serving different population groups were essential to efforts in New York City and San Diego. In the Minnesota refugee program, in which HBV testing was conducted during scheduled medical visits at a clinical facility, patient education, counseling, and navigation efforts appeared to be an effective strategy to ensure that persons who were HBsAg-positive attended a medical referral appointment. At the two community-based screening sites, however, ensuring that medical referral visits were attended was more challenging, and the rates of documented follow-up were lower than those of the Minnesota program.

What is already known on this topic?

In 2008, CDC recommended that all persons in the United States born in countries with a hepatitis B virus (HBV) infection prevalence ≥2% be tested and that those infected receive preventive counseling, education, and referral for medical management. Almost two thirds of persons with chronic HBV infection in the United States originated from countries with HBV infection prevalence ≥2%, but an estimated one half or fewer of these persons are aware of their infection, and a smaller proportion are receiving recommended medical monitoring and care. Efforts to increase the early identification of persons with chronic HBV infection and link them to medical care are a public health priority.

What is added by this report?

During October 2012–March 2014, in three sites participating in a CDC-funded initiative to identify foreign-born persons with HBV infection and link them to care, 4,727 persons were tested and 310 (6.6%) persons were HBsAg-positive. Rates of documented attendance at a follow-up visit were significantly higher for those referred from the refugee program (91%) than from community-based testing sites (33% and 56%). For all three sites, intensive patient counseling and assistance efforts were needed to achieve these results

What are the implications for public health practice?

Community-based and refugee clinic-based HBV testing initiatives can identify substantial numbers of persons with chronic HBV infection, inform them of their HBV infection, and provide preventive counseling. Strategies are needed to improve linkages from community-based testing sites to HBV-directed medical care.

The findings in this report are subject to at least three limitations. First, the three participating sites were not representative of all projects in the initiative, and persons born in East Asia, who are a substantial proportion of the foreign-born population originating in countries with intermediate to high HBV infection prevalence, were underrepresented (3). Second, data regarding attendance at scheduled medical visits were self-reported by patients, and difficulty in contacting these persons for follow-up might have resulted in underreporting. Finally, although HBsAg test results have high specificity (4), follow-up testing is recommended during medical evaluation to confirm chronic HBV infection status (1).

In cities with large populations of persons born in Asia and Africa, community-based efforts to screen foreign-born persons from countries with intermediate or higher HBV infection prevalence can identify substantial numbers of persons with chronic HBV infection, and persons who test HBsAg-positive can be successfully informed, counseled, and referred to medical care. Culturally and linguistically specific approaches were necessary in all phases of these initiatives. Outreach, counseling, and patient navigation activities in these populations require intensive effort and use of human resources. In refugee screening programs in which HBV testing is occurring, counseling and patient navigation might be effective in ensuring that those who are HBsAg-positive attend a first medical evaluation for chronic HBV infection. Linkage to a first medical visit from community-based testing venues is challenging, but can be accomplished with substantial patient navigation efforts. In this initiative, it was not possible to assess the quality or continuity of HBV-directed medical care for those persons who did attend a first referral appointment. Because routine and ongoing monitoring is the foundation for effective HBV medical management, future efforts to improve outcomes among foreign-born persons with chronic HBV infection should provide greater emphasis on this distal end of the process of “linkage to care” (5).

## Figures and Tables

**TABLE:** Results of programs at three selected sites funded by CDC to increase testing of foreign-born persons for hepatitis B and to link persons to medical care if they had positive hepatitis B surface antigen (HBsAg) test results, October 2012–February 2014

		HBsAg-positive									
		
				Received results		Counseled		Referred		Attended medical visit	
							
Project sites	Tested	No.	(%)	No.	(%)	No.	(%)	No.	(%)	No.	(%)
New York, New York	1,732	145	(8.4)	131	(90)	120	(83)	123	(85)	81	(56)
Minneapolis-St. Paul, Minnesota	1,800	117	(6.5)	111	(95)	111	(95)	106	(91)	106	(91)
San Diego, California	1,195	48	(4.0)	48	(100)	48	(100)	39	(81)	16	(33)
**Total**	**4,727**	**310**	**(6.6)**	**290**	**(94)**	**279**	**(90)**	**268**	**(86)**	**203**	**(66)**
